# Cardiovascular autonomic neuropathy in type 2 diabetes mellitus patients with peripheral artery disease

**DOI:** 10.1186/1758-5996-5-54

**Published:** 2013-09-25

**Authors:** Luís Henrique Canani, Eduardo Copstein, Miriam Pecis, Rogério Friedman, Cristiane Bauermann Leitão, Mirela Jobim Azevedo, Cristina Triches, Dimitris Rucks Varvaki Rados, Ruy Silveira Moreas, Jorge Luiz Gross

**Affiliations:** 1 Endocrine Division, Serviço de Endocrinologia do Hospital de Clínicas de Porto Alegre, Rua Ramiro Barcelos 2350, Prédio 12, 4º andar, Porto Alegre, Rio Grande do Sul, 90035-003, Brazil; 2 Cardiology Division of Hospital de Clínicas de Porto Alegre, Porto Alegre, Rio Grande do Sul, Brazil

**Keywords:** Diabetes, Diabetic neuropathy, Autonomic neuropathy

## Abstract

**Objective:**

To evaluate possible associations between cardiovascular autonomic dysfunction and peripheral artery disease (PAD) in patients with type 2 diabetes mellitus.

**Research design and methods:**

In this cross-sectional study, 67 patients with type 2 diabetes were included. PAD was identified by Doppler ultrasonography: systolic ankle-brachial pressure index <0.9. Cardiovascular autonomic function, besides five conventional cardiovascular autonomic function tests, was assessed by heart rate variability (HRV; 24-h ambulatory ECG recording) in time and frequency domains (spectral analyses) and three dimensional return maps. Power spectral analyses (PSA) were quantified in low frequency (LF), high frequency (HF), and very low frequency.

**Results:**

Patients with PAD (n = 30) had longer diabetes duration, higher systolic blood pressure (BP), waist-to-hip ratio, HbA1C test, and urinary albumin excretion (UAE) than patients without PAD. Most HRV indices in time domain were lower in patients with than without PAD. These patients also had lower PSA indices (LF=0.19±0.07 vs. 0.29±0.11 n.u.; LF/HF ratio=1.98±0.9 vs. 3.35±1.83; P<0.001) and indices of sympathetic (three-dimensional return map: P_1_-night 61.7±9.4 vs. 66.8±9.7; P=0.04) and vagal (24-h P_2_ 54.5±15.2 vs. 62.7±2.9; P< 0.02) activities (arbitrary units) than patients without PAD. Multivariate logistic regression analyses, adjusted for systolic BP, DM duration, HbA1C test, and UAE, confirmed the associations between impaired autonomic modulation and PAD, except for P_1_ index.

**Conclusion:**

In conclusion, patients with type 2 diabetes with PAD had lower HRV indices than patients without PAD, reflecting a dysfunction of cardiovascular autonomic modulation.

## Introduction

Patients with diabetes mellitus have a generalized atherosclerosis of the arterial bed, characterized by an early onset and a fast progression rate. Diabetes increases the risk for peripheral arterial disease (PAD) 2- to 3-fold [1]. Moreover, PAD, together with microvascular disease and peripheral neuropathy, is responsible for the high incidence of non traumatic amputations in patients with diabetes [1].

Intermittent claudication, the main clinical manifestation of PAD, has been associated with increased mortality [2]. The lower-extremity arterial calcification that occurs in PAD has been considered as a correlate of coronary artery calcification, at least in type 1 diabetes [3]. In patients with diabetes and PAD, besides the presence of coronary and cerebral atherosclerosis [4], cardiovascular autonomic neuropathy (CAN) can partially explain the high rate of cardiovascular mortality observed [5]. Disturbances in the autonomic system modulation seem to precede the increase in the thickness of the carotid intima, a predictor of atherosclerosis progression in patients with type 2 diabetes [6,7].

CAN has been traditionally diagnosed by cardiovascular autonomic function tests [8]. CAN can also be evaluated by heart rate variability (HRV) analyses [6], through the quantification of very small heart rate changes from one cardiac cycle to the next. This technology enables quantification of the relative influence of sympathetic and parasympathetic systems on the sinus node and, compared to conventional cardiovascular autonomic tests, has a higher sensitivity to detect autonomic abnormalities [9].

HRV detects both instantaneous heart rate and R-R intervals of the electrocardiogram. It is usually analyzed in time domain and frequency domain. The latter is also known as power spectral analysis (PSA). Time domain indices evaluate the dispersion of the cardiac R-R intervals around the mean, and reflect overall autonomic modulation on the sinus node. PSA evaluates the variance of R-R intervals as a function of frequency and is calculated by mathematical algorithms. The PSA results exhibits three main components: very low frequency (VLF; with no clearly defined participation in HRV), low frequency (LF; mainly sympathetic modulation), and high frequency (HF, parasympathetic modulation). PSA of HRV has been considered to be a useful tool in assessing the autonomic nervous system function in patients with diabetes [10].

A comprehensive evaluation of the autonomic nervous system function in diabetic patients with PAD has not been performed to date. This knowledge may contribute to earlier diagnosis and intervention in patients at higher cardiovascular risk in order to decrease the rate of lower extremity amputations and mortality. Therefore, the aim of the current study was to evaluate possible associations of cardiovascular autonomic dysfunction with PAD in patients with type 2 diabetes.

## Research design and methods

### Subjects

This was a cross-sectional study nested in a prospective cohort. Patients with type 2 diabetes were selected from a cohort of consecutive outpatients attending the Endocrine Division at Hospital de Clínicas de Porto Alegre. Details of the original cohort have been previously published [11]. From the original cohort of 98 patients with type 2 diabetes, 84 patients were available for evaluation. Missing patients (n = 14) did not differ from the included patients regarding age, diabetes duration, proportion of females, and ethnicity (data not shown).

The definition of type 2 diabetes was based on age of onset (>30 years old), no episodes of ketoacidosis and no need for insulin use during the first five years of diagnosis. Patients underwent a clinical and laboratory evaluation and the Ethics Committee approved the protocol. All patients gave written informed consent.

### General clinical evaluation

All patients answered a standard questionnaire that included questions about age, diabetes duration, smoking habit (defined as positive if the patient had smoked at least one cigarette a day for more than one year), and medications in use. Patients self-identified themselves as white or non-white (mixed or black). Patients were weighed in light clothes, without shoes, and height was recorded. Body mass index (BMI) was calculated as weight (kilograms) divided by squared height (meters). Waist and hip circumferences were measured with a soft tape in the upright position, on bare skin at the level the umbilicus and iliac crest, during mid-respiration, to the nearest 0.5 cm. Waist-to-hip ratio was calculated. Blood pressure (BP) was measured twice, in the sitting position, after a 10-min rest to the nearest 2 mmHg, using an aneroid sphygmomanometer. Hypertension was defined as BP levels ≥140/90 mmHg or the use of antihypertensive medication. Indirect ophthalmoscopy was performed by an ophthalmologist through dilated pupils and diabetic retinopathy was classified as present (any degree) or absent. The diagnosis of ischemic heart disease was based on the presence of at least one of the following: angina or possible infarct according to the WHO questionnaire for cardiovascular disease [12]; resting ECG abnormalities [Minnesota Codes: Q and QS patterns (1–1 to 1–3); S-T junction (J) and segment depression (4–1 to 4–4); T-wave items (5–1 to 5–3), and complete left bundle branch block (7–1)] [12], and/or perfusion abnormalities on myocardial scintigraphy at rest (fixed) or after infusion of dipyridamole. Based on their 24-h urinary albumin excretion (UAE), patients were classified as normoalbuminuric (<20 μg/min), microalbuminuric (20–199 μg/min) or macroalbuminuric (>199 μg/min) [13]. Diabetic nephropathy was diagnosed in the presence of micro- or macroalbuminuria and was always confirmed in a second urine sample collected over a 3- to 6-month period [13,14]. None of the patients with diabetic nephropathy had a doubtful etiology of their kidney disease (e.g. absence of retinopathy, rapid decline in glomerular filtration rate, resistant hypertension, or heavy proteinuria) [15]. Patients on angiotensin conversion enzyme inhibitors or angiotensin receptor antagonists had their urine samples collected after a minimum three-weeks interruption of these medications. The diagnosis of peripheral neuropathy was established if the Michigan score was greater than 2. This score is based on foot examination (deformities, callus, infection, ulceration), ankle reflex response, tactile sensitivity (normal, reduced or absent) and vibration perception threshold (normal, reduced or absent, 128 MHz tuning fork) at the dorsum of great toe [16]. The same trained examiner tested all participants.

### Peripheral artery disease assessment

PAD was accessed by systolic ankle-brachial BP ratio. The systolic BP was obtained on the ankle (posterior tibial and/or pedious arteries) and arms (brachial artery) of both sides, using Doppler ultrasonography [17]. The systolic BP ratio was calculated by dividing the ankle BP by the ipsilateral arm BP. PAD was defined by a ratio <0.9 on any side. Patients were divided into two groups according to systolic BP ratio values: with and without PAD.

### Autonomic nervous system evaluation

#### Cardiovascular autonomic function test

The autonomic nervous system was assessed by cardiovascular autonomic function tests as validated by Ewing and co-workers [8], and autonomic neuropathy was diagnosed when at least 2 out of 5 tests were abnormal [8,18]. Potentially interfering drugs were weaned off before the tests. No subject was on antiarrhythmic agents. Briefly, heart rate tests were performed with patients electrocardiographically monitored and heart rate was evaluated after 15 min of resting, before and after deep breath, during the Valsalva maneuver, and after standing. The BP tests evaluated the BP response one minute after standing up from lying down, and after sustained handgrip at 30% of the maximum voluntary contraction capacity, using a handgrip dynamometer.

#### Heart rate variability: time and frequency domain indices

HRV was also evaluated using ECG recording by the time domain and frequency domain methods (PSA), as well as the three dimensional return maps, during the day and the night periods [10]. The patients received verbal and written instructions to abstain from alcoholic and caffeinated beverages or caffeinated medications, and systemic decongestants, as well as to avoid vigorous exercises and not to wear elastic stockings from the day before the test. They were also advised to maintain their usual daily activities and to answer a questionnaire regarding the number of cigarettes smoked and any exceptional activity (extra physical activity or arguing) on the day of examination. For HRV analyses, ECG tapes were analyzed on a Mars 8000 (Marquete) scanner with a semiautomatic technique [10]. This software distinguishes normal beats from ectopic and artifacts and builds a time series of normal R-R intervals. Non sinus beats were eliminated and, for PSA, missing data were interpolated. PSA was computed for periods of 256 s using fast Fourier transformation. Only segments free of interpolations were analyzed.

The following time domain HRV indices were calculated from 24-h ECG recordings: mean of all R-R intervals, SD of the R-R intervals (SDNN), mean of the SD of R-R intervals calculated in 5-min segments (SDNNi), SD of averages of R-R intervals calculated in 5-min segments (SDANNi), root mean square of successive differences of adjacent R-R intervals (RMSSD), and percentage of differences between adjacent R-R intervals >50 ms (PNN50). The frequency-domain analyses were performed at complete rest, with patients lying in the supine position, in order to avoid loss of stationarity. During a 5-minute period, the following spectral components were assessed: total power (0.003-1 Hz), VLF (<0.04 Hz), LF (0.04-0.15 Hz), HF (0.15-0.5 Hz) and LF/HF ratio. LF component reflects both sympathetic and vagal tones, but mostly the sympathetic modulation, and HF component is closely related to respiratory frequency, reflecting vagal (parasympathetic) sinus node modulation. The total power and LF/HF ratio, which is a measure of sympathovagal balance, were calculated for each patient. The results of LF and HF were reported in absolute and normalized units (n.u.).

#### Heart rate variability: three-dimensional return map

The three-dimensional return map, a method based on nonlinear dynamics, reflects sympathetic and vagal modulation as we demonstrated by autonomic blockade studies with propranolol and atropine, in a reproducible way [10]. The map is constructed by plotting normal R-R intervals versus the difference between adjacent R-R intervals versus counts. Briefly, normal R-R intervals are plotted on the X-axis against the difference between adjacent R-R intervals on the Y-axis. Whenever superimposition of points occurs, the number of superimposed points is expressed on the Z-axis, normalized by the maximum density. A set of indices (P_1_, P_2_, P_3_, MN) is calculated to quantify the resulting three-dimensional images. P_1_ represents sympathetic modulation and is increased during sympathetic blockade with propranolol but is not affected by the administration of atropine. P_1_ is calculated as 100 minus the double of the mean slope between 10 and 90% of maximum density, in the plane that intersects the distribution in its maximum concentration of points, perpendicular to normal R-R intervals. P_1_ is inversely proportional to the mean slope of the distribution calculated at the maximum concentration of points. As concentration of points increase, the slope will be higher and P_1_ will be smaller. Therefore, the higher the sympathetic activity, the lower the value attributed to P_1_. P_2_ and P_3_ represent vagal modulation, are reduced during vagal blockade with atropine but are not affected by the administration of propranolol. To calculate P_2_ and P_3_, three-dimensional images are displayed as 10 equally spaced contour curves: P_2_ is calculated as the maximal longitudinal length, and P_3_, as the maximal transversal length of the outermost contour curve. The general index MN was calculated as the product of P_1_×P_2_×P_3_×10^-3^. MN represents either branches of the autonomic nervous system being affected by propranolol and atropine [10].

The 24-h ambulatory ECG recording and cardiovascular autonomic function tests were performed in the same day by the same investigator. Nighttime was recorded as the period between the time when the patient went to bed and the time when the patient woke up in the next morning.

### Laboratory evaluation

Urinary albumin excretion rate (UAER) was measured by immunoturbidimetry (Microalb; Ames-Bayer, Tarrytown, NY, USA; intra- and interassay coefficients of variations 4.5% and 11.0%, respectively). Glycated hemoglobin (HbA1c test) was measured by a high performance liquid chromatography (HPLC; Merck-Hitachi L-9100 glycated hemoglobin analyzer, reference range 4.7-6.0%; Merck Diagnostica, Darmstadt, Germany), fasting plasma glucose was measured by glucose-peroxidase colorimetric enzymatic method (Biodiagnostica, São Paulo, Brazil). Creatinine was measured by the Jaffé method and the lipid profile by a colorimetric method. Glomerular filtration rate was measured in 67 patients using the single-injection 51CrEDTA technique (coefficient of variation = 12%) [19].

### Statistical analyses

Variables are described as means ± standard deviation (variables with normal distribution), as median (range) (variables without normal distribution) and as the number of patients with the analyzed characteristic (percentage). Student’s t-test was used for continuous, normally distributed variables; Mann-Whitney’s U test was employed for non-normally distributed, continuous variables, and the chi-square test was used for categorical variables. There are no clearly established cutoff points to identify normal/abnomal values for some HRV indices. Therefore, we decided to categorize HRV indices according to their tertiles, based on studied sample values. This approach allows the identification of differences between groups since the lowest tertile for HRV indices should include the most abnormal values associated with CAN. To evaluate if the HRV indices (independent variables) were associated with PAD (dependent variable), multivariate logistic regression analyses were performed, adjusted for potential confounders. In these models, heart rate variability indices were categorized by tertiles and the upper (3^rd^) for each index was considered as the reference (OR = 1). HRV indices were log-transformed before analyses. P values <0.05 (two-tailed) were considered statistically significant. Statistical analyses were performed using SPSS 18.0 (SPSS, Chicago, IL).

## Results

Evaluation of CAN by HRV analyses was available in 67 out of 84 studied patients. The reasons for the exclusion from the protocol were as follows: incomplete tests (n = 10), presence of more than 10% of cardiac ectopic beatings on 24 h-ECG records, or cardiac arrhythmias that preclude the HRV analyses (n = 7). Frequency of PAD in the included patients was not different from all studied patients (35.8% vs. 35.7%; P = 0.881).

Clinical and laboratory characteristics of the 67 patients with and without PAD are depicted in Table 1. Patients with PAD had longer diabetes duration, higher waist-hip-ratio, and history of hypertension than those without PAD. Diabetic chronic complications, including autonomic neuropathy as evaluated by conventional cardiovascular autonomic function tests, were more frequent in patients with PAD as compared with patients without PAD. Regarding laboratory variables, the PAD group had a worse glycemic control and higher UAE than the group without PAD. There was no difference in diabetes treatment between patients with and without PAD.

**Table 1:** Clinical and laboratory characteristics of type 2 diabetic patients and the presence of peripheral arterial disease

	**With PAD**	**Without PAD**	**P**
n	24	43	-
Systolic ankle-brachial BP index	0.77 ± 0.09	0.98 ± 0.07	-
Age (years)	65.8 ± 6.8	63.2 ± 8.3	0.189*
Male	12 (50%)	16 (37%)	0.309δ
DM duration (years)	20.2 ± 7.1	15.2 ± 7.9	0.008*
Smoking	7 (29%)	10 (23%)	0.632^δ^
Body mass index (kg/m^2^)	30.1 ± 4.6	29.7 ± 4.8	0.773*
Waist-to- hip-ratio	0.98 ± 0.09	0.93 ± 0.08	0.059*
Diabetes treatment			
Oral agents	6 (25%)	20 (46%)	0.083^δ^
Insulin (with or without oral agents)	18 (75%)	23 (53%)	0.083δ
Insulin dose (U/day)	46.9 ± 19.8	46.2 ± 18.9	0.645*
Hypertension	23 (95%)	33 (76%)	0.043^δ^
Hypertension treatment			
ACE inhibitors	4 (16%)	14 (32%)	0.224^δ^
Diuretics	5 (21%)	12 (28%)	0.682^δ^
Calcium channel blockers	10 (42%)	14 (32%	0.498^δ^
Beta blockers	5 (21%)	4 (9%)	0.229^δ^
Peripheral neuropathy	17 (70%)	16 (37%)	0.011^δ^
Autonomic neuropathy	20 (83%)	28 (65%)	0.113^δ^
Fasting plasma glucose (mg/dl)	210.2 ± 70.5	176.3 ± 70.3	0.063*
HbA1C test (%)	7.9 ± 1.7	6.7 ± 1.6	0.010*
Total cholesterol (mg/dl)	222.6 ± 50.9	214.6 ± 45.2	0.511*
HDL cholesterol (mg/dl)	46.2 ± 26.9	45.6 ± 10	0.896*
Triglycerides (mg/dl)	164.5 (812)	134 (400)	0.097^**#**^
Serum creatinine (mg/dl)	1.1 ± 0.4	1.1 ± 0.9	0.858*
24-h UAE (μg/min)	90.3 (5560)	8.7 (2345)	0.000^**#**^

Data expressed as mean ± SD, median (range), or number of patients with the characteristic (%); PAD = peripheral arterial disease: BP = blood pressure.

Autonomic neuropathy: defined according to conventional autonomic cardiovascular function tests: two out five abnormal tests [8]. ^*^Student’s t-test; ^**#**^ Mann-Whitney’s U test; δ Chi-square test.

### Heart rate variability: time domain indices

**Figure 1:**
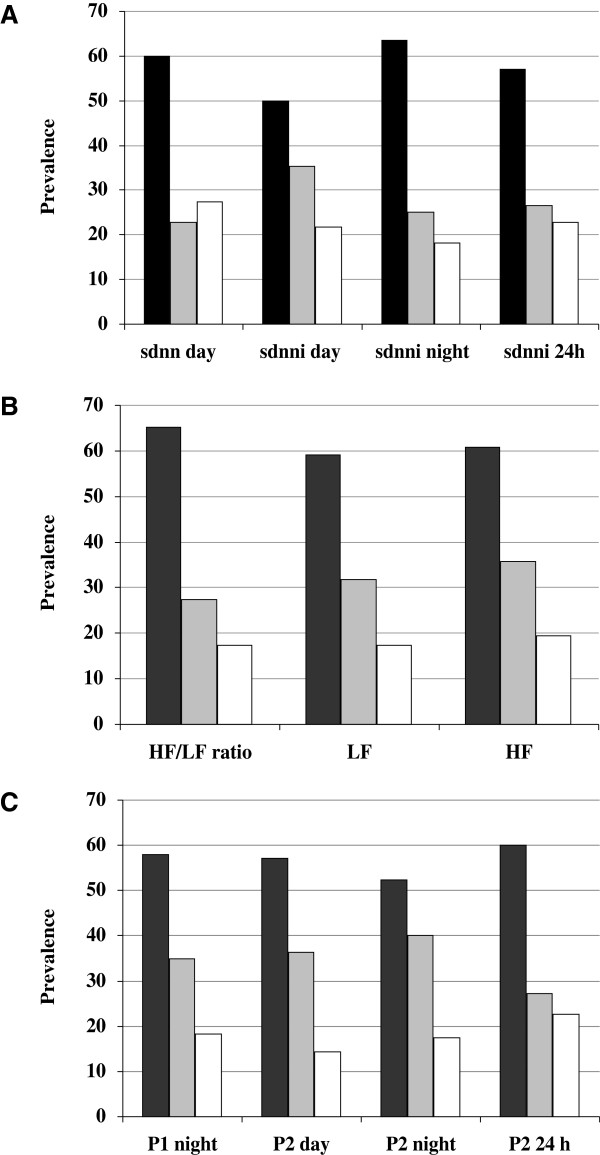
**Prevalence of peripheral artery disease according to autonomic neuropathy.** Prevalence of peripheral artery disease within each tertile for individual time domain indices **(A)**, power spectral analyses indices **(B)**, and Three Dimensional Return Map data **(C)**. Black bar represents first tertile, gray bar second tertile, and white bar third tertile. P<0.05 for all comparisons.

Regarding to time domain indices, patients with PAD had lower SDNN and SDNNi, at 24-h, day and night, and SDANN during the day as compared to patients without PAD (Table 2). Figure 1A shows the prevalence of PAD within each tertile for individual time domain indexes. PAD was more frequent in the 1^st^ tertile of SDNN during the day and of SDNNi at 24-h, day, and nigh.

**Table 2:** Heart rate variability analyses in time domain according to the presence of peripheral vascular disease

	**With PAD**	**Without PAD**	P^*^
n	24	43	-
Mean RR 24-h (ms)	767 ± 79	776 ± 92	0.98
Mean RR day (ms)	652 ± 263	738 ± 145	0.55
Mean RR night (ms)	811 ± 99	841 ± 93	0.41
SDNN 24-h (ms)	87 ± 35	105 ± 27	0.009
SDNN day (ms)	75.7 ± 34.2	92 ± 30,7	0.001
SDNN night (ms)	82.2 ± 39.1	96 ± 33.5	0.002
SDANN 24-h (ms)	3.54 ± 6.33	3.95 ± 5.6	0.16
SDANN day (ms)	3.42 ± 6.43	3.81 ± 0.6.3	0.02
SDANN night (ms)	3.86 ± 7.08	5.16 ± 7.7	0.13
SDNNi 24-h (ms)	18.4 ± 11.4	21.3 ± 12.9	0.002
SDNNi day (ms)	17.6 ± 11.7	20.1 ± 12.1	0.001
SDNNi night (ms)	19.7 ± 11.7	81.9 ± 37.5	0.004
RMSSD 24-h(ms)	28.5 ± 11.8	38.3 ± 13.7	0.16
RMSSD day (ms)	27.3 ± 11.3	36.9 ± 13.8	0.21
RMSSD night (ms)	30.6 ± 14.2	42.2 ± 16.1	0.15
PNN50 24-h (%)	76.4 ± 29.3	87.8 ± 25.9	0.19
PNN50 day (%)	63.4 ± 29.9	76.7 ± 26.6	0.23
PNN50 night (%)	67.7 ± 31.5	73.0 ± 27.5	0.22

Data were log transformed before analyses and then expressed as mean ± SD.

PAD = peripheral arterial disease; SDNN = SD of the R-R intervals, SDNNi = mean of the SD of R-R intervals calculated in 5-min segments, SDANNi = SD of the averages of the R-R intervals calculated in 5-min segments; RMSSD = root mean square of successive differences of adjacent R-R intervals; (PNN50) = percentage of differences between adjacent R-R intervals >50 ms;

^*^ Student’s t-test.

### Heart rate variability: frequency domain indices

In the analyses of HRV in frequency domain (Table 3), total spectral power, VLF, LF and HF components, were lower in patients with PAD as compared to patients without PAD. Figure 1B shows the frequency of PAD within each tertile for individual PSA indices. PAD was more frequent in the 1^st^ tertile of LF, HF and LF/HF ratio.

**Table 3:** Heart rate variability analyses in frequency domain (power spectral analyses) and three dimensional return map data of type 2 diabetic patients according to peripheral arterial disease

	**With PAD**	**Without PAD**	P*
	**n = 24**	**N = 43**	
**Power spectral analyses **^**#**^			
Very low frequency ms^2^/Hz	328 ± 295	755 ± 924	0.032
Low Frequency ms^2^/Hz	123 ± 152	490 ± 815	0.01
High Frequency ms^2^/Hz	61 ± 85	137 ± 162	0.005
Total Power ms^2^/Hz	538 ± 513	1406 ± 1880	0.031
Low frequency (LF) n.u.	0.55 ± 0.13	0.75 ± 0.42	0.56
High frequency (HF) n.u.	0.31 ± 0.06	0.25 ± 0.14	0.46
Low frequency/ High frequency	1.98 ± 0.9	3.37 ± 1.82	0.067
**Three dimensional return map**			
P_1_ 24-h	59.5 ± 9.5	61.6 ± 8.7	0.38
P_1_ day	58.0 ± 9.5	61.0 ± 7.7	0.17
P_1_ night	61.7 ± 9.4	66.8 ± 9.7	0.04
P_2_ 24-h	54.5 ± 15.2	62.7± 2.9	0.02
P_2_ day	46.5 ± 13.6	54.9 ± 12.7	0.01
P_2_ night	44.5 ± 14.5	51.9 ± 12.7	0.03
P_3_ 24-h	75.0 ± 37.1	80.3 ± 30.8	0.52
P_3_ day	65.4 ± 33.7	72.5 ± 30.3	0.37
P_3_ night	61.4 ± 24	65.6 ± 23	0.47
MN 24-h	272 ± 227	326 ± 195	0.31
MN Day	195 ± 156	259 ± 182	0.15
MN night	186 ± 136	247 ± 135	0.08

Data expressed as mean ± SD; P_1_ = index reflecting sympathetic modulation; P_1_, P_2_, and P_3_ are expressed as arbitrary units; P_2_ and P_3_ = indices reflecting vagal modulation; MN = global modulation; ^*^ Student t-test; ^**#**^ Data were log transformed before analyses.

### Heart rate variability: three-dimensional return map

The three dimensional return map data (Table 3) shows sympathetic (P_1_), vagal (P_2_ and P_3_) and global modulation (MN) during the 24-h, day, and night periods. P_1_ was lower during the night and P_2_ was lower at all periods in patients with PAD in comparison with those without PAD. When P_1_ and P_2_ were stratified into tertiles, patients in the lowest tertiles had the highest frequency of PAD (Figure 1C).

### Multivariate analyses

Logistic regression analyses were performed to evaluate the association of PAD (dependent variable) with tertiles of HRV indices, taken into account possible confounders, namely, diabetes duration, glycemic control, triglycerides, and UAE (Table 4). The 1^st^ tertile of SDNN at day and SDNNi at night increased the chance of PAD being present 6.08 (CI 95% 1.24-29.9; P = 0.026) and 7.67 times (CI 95% 1.6-37.3; P = 0.012), respectively. Having a high frequency index or a low frequency index in the lowest tertiles respectively raised the chance of PAD 7.37 times (CI 95% 1.5-37.3; P = 0.016) and 6.17 times (CI 95% 1.1-35.1; P = 0.04). The 1^st^ tertile of HF/LF ratio was also associated with an increased chance for the presence of PAD (OR 7.42, CI 95% 1.5-37.1; P = 0.015). Regarding the three dimensional return maps analyses, only a daily vagal modulation index (P_2_) in the lowest tertile increased the chance for PAD 8.42 times (CI 95% 1.5-47.5; P = 0.016). To take into account a possible effect of fasting plama glucose (FPG) at the start of the test, the same multivariate logistic regression analyses models were performed including FPG instead of HbA1C. No significant differences were observed in the main results (data not shown).

**Table 4:** Multivariate logistic regression analyses: heart rate variability indices (independent variables) categorized by tertiles and their respective odds ratios for the presence of peripheral arterial disease (dependent variable)

	Odd ratio*	**95%CI**	**P**
**Time domain indices**			
**SDNN day**			
1^st^ tertile	6.08	1.24 - 29.90	0.026
2^nd^ tertile	0.80	0.20 - 3.90	0.784
3^rd^ tertile	1.0	-	-
**SDNNi night**			
1^st^ tertile	7.67	1.60 - 37.30	0.012
2^nd^ tertile	1.14	0.20 - 6.20	0.878
3^rd^ tertile	1.00	-	-
**Frequency domain indices**			
**High Frequency**			
1^st^ tertile	7.37	1.50 - 37.30	0.016
2^nd^ tertile	5.16	0.90 - 30.00	0.068
3^rd^ tertile	1.00	-	-
**Low Frequency**			
1^st^ tertile	6.17	1.10 - 35.10	0.040
2^nd^ tertile	4.00	0.70 - 24.40	0.129
3^rd^ tertile	1.00	-	-
**High Frequency / Low Frequency Ratio**			
1^st^ tertile	7.42	1.50 - 37.10	0.015
2^nd^ tertile	2.01	0.40 - 11.30	0.392
3^rd^ tertile	1,00	-	-
**Tridimensional return map**			
**P**_**1 **_**night**			
1^st^ tertile	2.67	0.60 - 11.80	0.195
2^nd^ tertile	0.73	0.10 – 4.10	0.720
3^rd^ tertile	1.00	-	-
**P**_**2 **_**day**			
1^st^ tertile	8.42	1.50 - 47.50	0.016
2^nd^ tertile	2.59	0.40 - 15.90	0.305
3^rd^ tertile	1.00	-	-

* the odds ratio was adjusted for diabetes duration, HbA1C, hypertension, serum triglycerides, and urinary albumin excretion in each model. SDNN= SD of the R-R intervals; SDNNi = mean of the SD of R-R intervals calculated in 5-min segment; P_1_ = index reflecting sympathetic modulation; P_2_ = index reflecting vagal modulation; upper index tertile (3^rd^) for each index was considered as the reference (OR = 1).

## Discussion

In this sample of patients with type 2 diabetes, PAD was associated with autonomic neuropathy, as evaluated both by conventional cardiovascular autonomic function tests and HRV indices. This association remained even after adjustments for glycemic control, triglycerides, hypertension, diabetes duration, and UAE. Moreover, the HRV analyses allowed the evaluation of sympathetic/parasympathetic balance in these patients.

The association of autonomic nervous system with PAD was confirmed in the multivariate analyses for some of the time domain indices and frequency domain indices including the HF/LF ratio. This means that there was an abnormal modulation of both vagal and sympathetic systems. Although the role of time and frequency domain indices in the evaluation of the autonomic nervous system has been widely debated, autonomic blockade studies confirm that these indices represent mostly vagal modulation of the sinus node [20]. Furthermore, in the multivariate analyses only the daily P_2_ index, which represents the parasympathetic component of HRV, was associated with PAD. In summary, we demonstrated an association of PAD and abnormal autonomic nervous system and our data suggest that the most affected branch was the parasympathetic.

In the current study, day and night periods were analyzed separately. In normal subjects there is a circadian pattern of heart rate autonomic modulation with a reduced HRV during the day, secondary to increased sympathetic activity, and an increased HRV during the night due to the predominance of vagal modulation. In fact we demonstrated reduced HRV time and frequency domain indices at day and night and an abnormal P_2_ during the day only.

The association between PAD and autonomic neuropathy could be explained by the insulin resistance observed in patients with type 2 diabetes. In The Edinburgh Artery Study, a higher prevalence of PAD was observed in patients with diabetes in comparison with those without it, probably due to the higher systolic BP and triglycerides observed in these patients, both variables associated with insulin resistance [21]. Similarly, insulin resistance has been associated with low HRV [22], reinforcing the idea that hyperinsulinemia could be associated with an increased sympathetic tonus [23]. In the present study, a higher prevalence of hypertension and a larger waist-to-hip ratio were observed in patients with PAD, parameters that have been associated with insulin resistance. However, in the current study the association of HRV abnormalities with PAD was independent of blood pressure, as demonstrated in the multivariate analyses. This result suggests a direct association of autonomic dysfunction with PAD.

A relationship of atherosclerosis with autonomic cardiovascular dysfunction has already been observed [24]. However, as far as we known, no study demonstrated the association of PAD with CAN. The EURODIAB data showed an association of low HDL cholesterol and high triglycerides in patients with cardiovascular autonomic neuropathy, suggesting a role for an adverse lipid profile in the pathogenesis of CAN [25]. Autonomic neuropathy was already related to lower-extremity arterial calcification in type 1 diabetes [3]. In patients with type 2 diabetes the LF index, a HRV parameter that reflects mainly sympathetic modulation, predicted progression of carotid atherosclerosis [7] and coronary artery disease [6,24,26]. None of these studies evaluated the association of HRV abnormalities with PAD.

Possible limitations of the present study are related to the accuracy of HRV indices to evaluate sympathetic modulation. Maybe the use of controlled sympathetic stimulation - as head up tilt or mental stress - could add more information about the cardiac sympathetic modulation in these patients. Another factor that could have reduced the detection of HRV abnormalities in the current study was the exclusion of patients with arrhythmias since this group possibly had even lower HRV. However, despite these methodological limitations, the consistent demonstration of differences in various HRV indices between patients with and without PAD suggests that the results of the present study are true.

## Conclusion

In this sample of patients with type 2 diabetes, those with PAD had lower HRV indices than patients without PAD, reflecting a dysfunction of cardiovascular autonomic modulation. This impaired cardiac autonomic modulation might represent an additional cardiovascular risk factor for patients with type 2 diabetes and PAD. However, the role of these abnormalities as predictors of mortality in diabetic patients with PAD should be evaluated in prospective studies.

## Competing interests

The authors declare that they have no competing interests.

## Authors’ contributions 

Conceived and designed the study: JLG and LHC. Performed the subjects’ clinical evaluation: EC, CT, and MP. Evaluated the autonomic function tests: RSMF. Analyzed the data: LHC, EC, CBL RF, and DRVR. Drafted the manuscript: LHC, EC, RF, RSMF, MJA, and JLG. Wrote the final version of the paper and answers the reviewers’ queries: LHC, CBL, MJA, DRVR, and RSMF. Coordinated the study: JLG and LHC. All authors read and approved the final manuscript.
